# Oligomeric state of the aspartate:alanine transporter from *Tetragenococcus halophilus*

**DOI:** 10.1093/jb/mvac057

**Published:** 2022-07-11

**Authors:** Akari Miyamoto, Takashi Yamanaka, Satomi Suzuki, Kota Kunii, Kenichiro Kurono, Akira Yoshimi, Masafumi Hidaka, Satoshi Ogasawara, Kei Nanatani, Keietsu Abe

**Affiliations:** Laboratory of Applied Microbiology, Department of Microbial Biotechnology, Graduate School of Agricultural Science, Tohoku University, Sendai, Miyagi, 980-0845 Japan; Laboratory of Applied Microbiology, Department of Microbial Biotechnology, Graduate School of Agricultural Science, Tohoku University, Sendai, Miyagi, 980-0845 Japan; Laboratory of Applied Microbiology, Department of Microbial Biotechnology, Graduate School of Agricultural Science, Tohoku University, Sendai, Miyagi, 980-0845 Japan; Laboratory of Applied Microbiology, Department of Microbial Biotechnology, Graduate School of Agricultural Science, Tohoku University, Sendai, Miyagi, 980-0845 Japan; LS-Project, Shoko Science Co., Ltd., Aoba-ku, Yokohama, 225-0012 Japan; Microbial Genomics Laboratory, New Industry Creation Hatchery Center; Laboratory of Molecular Enzymology, Department of Molecular Cell Science, Graduate School of Agricultural Science, Tohoku University, Sendai, Miyagi, 980-0845 Japan; Department of Chemistry, Graduate School of Science, Chiba University, Chiba, 263-8522 Japan; Molecular Chirality Research Center, Chiba University, Chiba, 263-8522 Japan; Membrane Protein Research and Molecular Chirality Research Centers, Chiba University, Chiba 263-8522, Japan; Laboratory of Applied Microbiology, Department of Microbial Biotechnology, Graduate School of Agricultural Science, Tohoku University, Sendai, Miyagi, 980-0845 Japan; Structural Biology Group, Advanced Research Center for Innovations in Next-Generation Medicine, Tohoku University, Sendai, Miyagi, 980-8573, Japan; Laboratory of Applied Microbiology, Department of Microbial Biotechnology, Graduate School of Agricultural Science, Tohoku University, Sendai, Miyagi, 980-0845 Japan; Microbial Genomics Laboratory, New Industry Creation Hatchery Center

**Keywords:** SEC-MALS, blue native PAGE, AspT, amino acid transporter

## Abstract

The aspartate:alanine exchanger family of membrane transporters includes industrially important transporters such as succinate exporter and glutamate exporter. No high-resolution structure is available from this family so far, and the transport mechanism of these transporters also remains unclear. In the present study, we focus on the oligomeric status of the aspartate:alanine antiporter (AspT) of *Tetragenococcus halophilus*, which is the prototype of this family. To investigate the oligomeric structure of AspT, we established a system that produces high yields of highly purified AspT and determined the oligomeric structure of AspT by analysis with size exclusion chromatography coupled with multi-angle light scattering and blue native PAGE and by comparison of the wild-type AspT with a single-cysteine mutant that forms spontaneous inter-molecular thiol crosslinking. All the results consistently support the notion that AspT is a homodimer in solutions and in membranes.

## Graphical Abstract

**Graphical Abstract:**
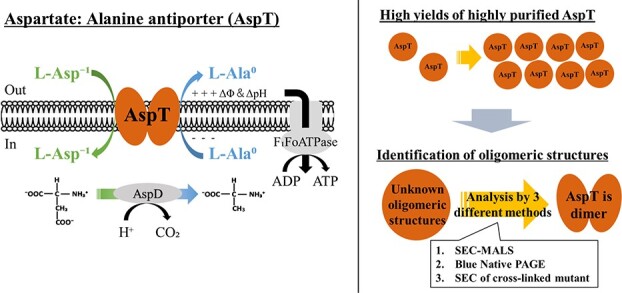

## Abbreviations

AAEx: aspartate:alanine exchanger
AspT: aspartate:alanine antiporter
BN: blue native
DDM: *n*-Dodecyl-*β*-D-maltopyranoside
DLS: Dynamic Light Scattering
MALS: multi-angle light scattering
SEC: size exclusion chromatography
TTBS: Tween Tris-buffered saline pH 7.4
WT: wild type

## Introduction

Previous research on improving production efficiency in fermentation has indicated the important roles played by a mechanosensitive channel (NCgl1221) in L-glutamate overproduction from *Corynebacterium glutamicum* (1) and by substrate-uptake transporters in ethanol fermentation from *Saccharomyces cerevisiae* (2). These and similar studies make it clear that substrate uptake and product export are critical processes for effective chemical production via microbial fermentation. Currently, biotechnological research is developing practical ways to regulate these forms of transport, and a protein of high interest is the aspartate:alanine antiporter (AspT), which was originally found in the lactic acid bacterium *Tetragenococcus halophilus* and is a member of the aspartate:alanine exchanger (AAEx) family of transporters (Transporter Classification Database #2.A.81). AspT catalyses the import of L-aspartate from media and the export of L-alanine to outside the cell (3, 4); our group has extensively studied its topology and functions (5–7).

AspT consists of 543 amino-acid residues (Mass/kDa 57.2). AspT has a unique topology of 10-transmembrane helices assembled with two re-entry helices between transmembrane helices 4–5 and 9–10 (6, 7). It has been demonstrated that inverted repeats in several membrane transporters play important mechanistic roles in alternating-access mechanisms (8, 9). Such type of arrangement in membrane domains was recently reported in the Na^+^-dependent citrate symporter of *Klebsiella pneumoniae* (CitS) (10).

Other transporters important for industrial use, such as the succinate exporter SucE1 from *C. glutamicum* (11), also belong to the AAEx family; therefore, knowledge of the substrate transport mechanism of AspT will contribute to the application of other AAEx transporters in the food, chemical and pharmaceutical industries. To use AspT and other AAEx family members for industrial purposes, it is essential to develop new technologies that regulate their substrate transport activities and substrate specificity. To this end, structural and functional information about the target transporter proteins is indispensable for protein engineering, and the molecular structure of AAEx family transporters needs to be elucidated to clarify their functional mechanisms. So far, we have reported some functional properties and topological information about AspT (6, 7, 12). Although Nanatani *et al*. (6) previously demonstrated that AspT formed oligomers by crosslinking with glutaraldehyde, the oligomeric structure was unclear. In the present study, we established a system that can produce a high yield of highly purified AspT and confirmed the transport function of the purified AspT by reconstitution in liposomes. Then, we analysed the oligomeric structure of purified AspT by means of size exclusion chromatography (SEC) coupled with multi-angle light scattering (MALS) and by blue native (BN)-PAGE; furthermore, the dimeric status was confirmed by comparison with a previously identified single-Cys mutant that forms spontaneous inter-molecular disulphide bonds.

## Materials and Methods

### Bacterial strains and culturing

*Escherichia coli* XL1-Blue (*recA1 endA1 gyrA96 thi-1 hsdR17 supE44 relA1 lac* [F’*proAB lacI^q^ZM15* Tn10 (Tet^r^)]) harbouring pMS421 (*Spec^r^*, *lacI^q^*), referred to as strain XL3 (3), *E. coli* C43 (DE3) (F^−^*ompT hsdSB* (*rB^−^ mB^−^*) gal dcm (DE3)) (Lucigen Corporation, Wisconsin, CA) and *E. coli* C43 (DE3) pLysS (Lucigen Corporation) were used to express histidine-tagged AspT. The pTrc99A (13) plasmid containing the asp operon (referred to as pTrcAspD/T-His; the hexa-histidine tag was inserted into loop 5 of AspT) was used (7).

*Escherichia coli* strains XL3, C43 (DE3) and C43 (DE3) pLysS harbouring plasmid pTrcAspD/T-His were cultured with Luria Bertani (LB) medium. After addition of 0.2 mM isopropyl-β-D-thiogalactoside (IPTG), the cells were incubated under static (0 rpm) or shaking (80 or 160 rpm) conditions at 37°C for 12 h. After IPTG induction, the cells were centrifuged (4,620 × *g*, 10 min, 4°C) and collected. After re-suspension in 0.1 M potassium phosphate buffer (K-Pi), cells were centrifuged again under the same conditions, the supernatant was removed, and the wet weight of the bacteria was measured. Whole protein was extracted from collected cells with SDS sample buffer and subjected to SDS-PAGE.

### western blotting

Purified wild-type (WT) AspT and a mutant, L60C, were each suspended at a concentration of 0.2 μg/μl in the same amount of sample buffer (62.5 mM Tris–HCl (pH 6.8), 2% SDS, 10% glycerol, 0.005% bromophenol blue) either with 5% 2-mercaptoethanol (for the reducing condition) or without (for the non-reducing condition) and incubated at 37°C for 30 min. Aliquots of 0.5 μg of purified protein were subjected to SDS-PAGE (14). After electrophoresis, the gels were stained with Coomassie Brilliant Blue Stain One Super (Nacalai Tesque Inc., Kyoto, Japan).

For western blotting, the proteins were transferred to PVDF membranes (Immobilon-P, Merck Millipore, Burlington, MA). The PVDF membranes were blocked with blocking buffer (2% skim milk, 25 mM Tris–HCl (pH 7.4), 137 mM NaCl, 3 mM KCl, 0.05% Tween20) for 30 min at room temperature and incubated overnight with a His-tag antibody (Medical & Biological Laboratories Co., Nagoya, Japan) diluted 5000-fold in Tween Tris-buffered saline pH 7.4 (TTBS). After washing, the PVDF membranes were incubated with horseradish peroxidase-conjugated anti-mouse antibody (Promega Co., Madison, WI, USA) diluted 5000-fold in TTBS for 30 min at room temperature. The antibody reactions were detected with SuperSignal™ West Pico PLUS Chemiluminescent Substrate (Thermo Fisher Scientific, Waltham, MA) by using an imaging system (LAS-4000 mini, GE Healthcare, Chicago, IL).

### Expression and purification, transport assay of AspT

*Escherichia coli* cells harbouring the pTrcAspD/T-His plasmid were pre-cultured in 2× LB medium at 30°C for 25 h. The pre-cultured cells (4.15 ml) were transferred to 166 ml of fresh LB medium containing 30 mM D-glucose, 30 μg/ml carbenicillin, 1 mM pyridoxal 5′-phosphate and 50 mM L-aspartate (K salt, pH 7.0), and incubated with shaking at 37°C. When the absorbance at 660 nm reached roughly 0.4, 0.2 mM (final concentration) IPTG was added to the medium. To select for transformants and maintain plasmids, 30 μg/ml carbenicillin, 30 μg/ml spectinomycin and 25 μg/ml chloramphenicol were used. After a 12-h induction, the bacteria were harvested by centrifugation (4,620 × *g*, 10 min, 4°C). A portion of the cells was treated with SDS sample buffer at 37°C for 30 min and subjected to polyacrylamide gel electrophoresis. AspT expression levels in the harvested cells were detected by western blotting with anti-AspT antibodies (6). The statistical significance of the expression level and cell yield was analysed with Bonferroni multiple comparisons to compare the three subject groups with each other. Significance was defined as a *P* < 0.05.

Solubilization and purification of AspT were carried out following the methods of Sasahara *et al*. (5) and Suzuki *et al*. (12). In brief, the collected cells were disrupted by a high-pressure homogeniser (EmulsiFlex B15 from Avestin Inc., Ottawa, Canada) and ultracentrifuged (193,011 × *g*, 1 h, 4°C) to obtain membrane fractions. For cell disruption, 0.1 M K-Pi buffer (pH 7.0) containing 1 mg/ml (final concentration) lysozyme and 50 ng/ml DNase I was used as the cell suspension buffer. Membrane fractions were solubilized with 1.5% *n*-Dodecyl-*β*-D-maltopyranoside (DDM), and AspT was purified with TALON Metal Affinity Resin (Clontech Laboratories, Inc., Mountain View, CA, USA). The purified AspT was subjected to SDS-PAGE. The protein concentration of purified AspT was quantified by ImageJ (15, 16) using bovine serum albumin for the standard curve. To optimize the lysozyme concentration during cell disruption, 0.1 M K-Pi buffer (pH 7) containing 0, 0.01, 0.1 or 1 mg/ml (final concentration) lysozyme and 50 ng/ml DNase I was used as the cell suspension buffer. Reconstitution of AspT into liposomes and the subsequent transport assay were carried out by following the method of Suzuki *et al*. (12).

### Size-exclusion chromatography

After ultracentrifugation at 190,000 × *g* for 30 min at 4°C, 0.05 μg of the purified AspT was analysed with the Prominence UFLC system (Shimadzu Co., Kyoto, Japan) using a Superdex 200 Increase 10/300 GL column (Cytiva, Marlborough, MA, USA) and a running buffer of 50 mM HEPES-HCl (pH 7) containing 0.01% DDM, 500 mM NaCl and 200 mM L-Aspartate (K-salt) at a flow rate of 0.8 ml/min. Tryptophan fluorescence (Ex, 325 nm; Em, 280 nm) of the eluate was monitored by using a fluorescence detector (L-7485) (Hitachi Science & Technology, Tokyo, Japan).

### Dynamic light scattering analysis

The particle size of AspT-DDM complex in a general buffer of 20 mM HEPES-NaOH (pH 7.0), 500 mM NaCl, 200 mM Asp-KOH (pH 7.0), 0.01% DDM and 20% glycerol was analysed by dynamic light scattering (DLS) analysis in a DynaPro Nanostar system (Wyatt Technology Co., Santa Barbara, CA, USA) with 95 mW HeNe laser, 658 nm λ at 25°C. An aliquot of 10 μl of solubilized AspT (2.1 mg/ml) was measured by DLS three times.

### SEC-MALS analysis

The purified AspT was further subjected to SEC with a Superdex 200 pg 16/600 column (Cytiva) (4°C; flow rate 1 ml/min) and fractionated. The SEC-purified AspT was concentrated to 2.4 mg/ml of AspT using an Amicon Ultra-0.5 filtration device (30 kDa) (Merck Millipore). To remove extra free DDM micelles and the exchange buffer, the concentrated AspT fraction was dialysed with the buffer used in the DLS analysis using a Spectra-Por® Micro Float-A-Lyzer® Dialysis Device MWCO 100 kDa (Repligen Co., Waltham, MA, USA). In order to determine the absolute molecular mass of a functional unit of AspT, we performed SEC-MALS analysis using a Superdex 200 Increase 10/300 GL column (Cytiva) and a Prominence HPLC system (Shimadzu Corp.) with an MALS detector DAWN HELEOSII (Wyatt Technology), a RI detector Optilab T-rEX (Wyatt Technology) and a UV detector SPD-20A (Shimadzu Corp.) run in the buffer used in the DLS analysis, with a flow rate of 0.8 ml/min.

### BN-PAGE analysis

BN PAGE analyses of AspT were performed using a NativePAGE™ Bis-Tris Gel System (Thermo Fisher Scientific) according to the manufacturer’s instructions. NativePAGE™ 3–12% Bis-Tris Gel 1.0 mm × 10 well (Thermo Fisher Scientific) was used for gradient gels. A 4-μl aliquot of purified AspT (0.7 mg/ml) or one of the following molecular weight markers: 4 μl catalase (1 mg/ml); 4 μl aldolase (1 mg/ml); or 2 μl 6-phosphogluconate dehydrogenase (1.7 mg/ml), mixed with 2.5 μl of NativePAGE™ 4X Sample Buffer (Thermo Fisher Scientific); and 2 μl of NativePAGE™ 0.05% G-250 Sample Additive (Thermo Fisher Scientific), and ultra-pure water was added to bring the volume to 10 μl. The resultant samples were applied to BN-PAGE and electrophoresed at 100 V for 3 h at room temperature. After electrophoresis, gels were stained using a NativePAGE Novex Bis-Tris Gel System (Thermo Fisher Scientific) following instructions in the manual. Gel images were scanned by a GT-X820 image scanner (Seiko Epson Corp., Nagano, Japan).

### Purification of the AspT WT and L60C mutant for SEC

Expression of L60C mutants was in accordance with Nanatani *et al.* (6). Membrane fractions obtained from WT and mutant L60C cells in 1 l of culture were incubated for 1 h at 4°C in 4 ml of a solubilization buffer (consisting of 20 mM Na-Pi (pH 6.0), 200 mM NaCl, 500 mM Asp-NaOH (pH 6.0), 20% glycerol, and 1 mM PMSF) and containing 1.5% DDM. The mixture was shaken at 4°C for 1 h. After solubilization, the samples were ultracentrifuged at 193,000 × *g* and 4°C for 35 min using a 50.2 Ti XL-90 rotor (Beckman Coulter, Inc., Brea, CA, USA). The supernatant after ultracentrifugation was mixed with equilibrated Ni Sepharose® Excel (Cytiva), 2 mM imidazole was added, and the mixture was incubated for 1 h at 4°C.

After adsorption, the solution was poured into Econo-Pac® Chromatography Columns (Bio-Rad Laboratories, Inc., Hercules, CA, USA) and washed four times with the solubilization buffer containing 0.02% DDM and 5 mM imidazole. After washing, AspT or L60C was eluted with the solubilization buffer (without PMSF) containing 0.02% DDM and 250 mM imidazole.

Each protein solution of affinity-purified WT and L60C was concentrated using ultrafiltration membranes (Ultra-10 Centrifugal Filters Ultracel®-100 K, Merck, Darmstadt, Germany). The concentrated solution was subjected to gel filtration chromatography using a Superdex 200 Increase 10/300 GL column (Cytiva) and an elution buffer of 20 mM Na-Pi (pH 6.0) containing 500 mM NaCl, 200 mM Asp-NaOH (pH 6.0) and 0.01% DDM at a flow rate of 0.4 ml/min. UV absorbance at 280 nm was monitored using a UV detector (AKTA Purifier 10, Cytiva).

**Fig. 1:**
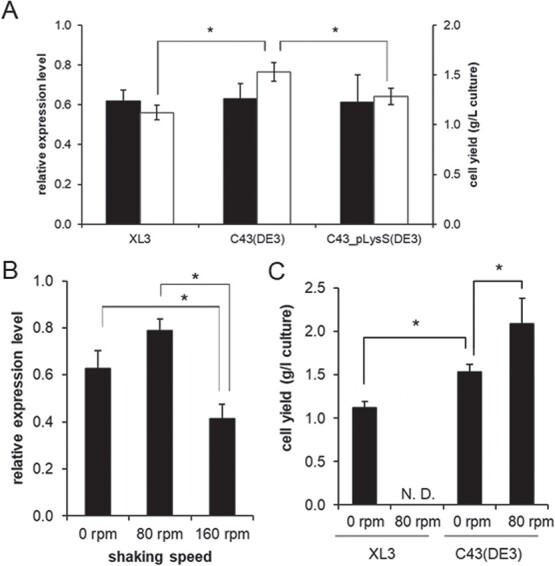
**Optimization of *E. coli* host strains and culture conditions for AspT overexpression.** (A) *E. coli* strains XL3, C43 (DE3) and C43 (DE3) pLysS harbouring plasmid pTrcAspD/T-His were cultured with LB medium. After addition of 0.2 mM IPTG, the cells were incubated at 37°C under static conditions for 12 h. Whole protein was extracted from collected cells with SDS sample buffer and subjected to SDS-PAGE. AspT expression was detected by western blotting using an anti-AspT peptide polyclonal antibody. Relative expression levels for each strain were normalized to 0.05 μg of purified AspT, as indicated by the black bars (vertical axis on the left). The cell yields for each strain from 1 l of culture medium are shown as white bars (vertical axis on the right). (B) *E. coli* C43 (DE3) harbouring plasmid pTrcAspD/T-His was cultured with LB medium. After addition of 0.2 mM IPTG, the cells were incubated under static (0 rpm) or shaking (80–160 rpm) conditions at 37°C for 12 h. AspT expression was detected by western blotting using an anti-AspT peptide polyclonal antibody, and relative expression levels are presented as described above. (C) *E. coli* strains XL3 and C43 (DE3) harbouring plasmid pTrcAspD/T-His were cultured with LB medium. After addition of 0.2 mM IPTG, the cells were incubated under static or shaking (80 rpm) conditions at 37°C for 12 h. The cell yields from 1 l of culture medium under each condition are shown. Because *E. coli* XL3 cells were lysed under the 80-rpm shaking condition, the cell yield for this condition is recorded as nondetectable (N.D.). (A–C) Data are presented as the means of three independent experiments. Error bars indicate S.D. (^*^P < 0.05).

## Results

Analysis of the oligomeric structure of AspT requires a large amount of purified protein. However, as a membrane protein, AspT is very difficult to express in high yields and is also difficult to purify sufficiently for analysis with conventional AspT expression and purification methods. Therefore, we reconstructed the expression and purification methods of AspT. First, to find an appropriate host for AspT overexpression, *E. coli* strains XL3, C43 (DE3) and C43 (DE3) pLysS harbouring pTrcAspD/T-His were cultured in LB medium. After IPTG induction for 12 h, the cells were harvested and treated with SDS sample buffer. Five micrograms of extracted protein from whole cells were subjected to SDS-PAGE, and AspT expression was detected by western blotting with an anti-AspT polyclonal antibody (6).

No clear differences in AspT expression levels were observed among the three strains (Fig. 1A, black bars), indicating that the expression of AspT is strain independent. On the other hand, yields of total cells showed a slight difference among the three strains (Fig. 1A, open bars). *Escherichia coli* C43 (DE3)/pTrcAspD/T-His showed the highest cell yield (1.53 ± 0.09 g/l culture), and we selected the C43 (DE3) strain as our AspT expression host. Then, we optimized the culture conditions for AspT expression with *E. coli* C43 (DE3)/pTrcAspD/T-His. The shaking speed of each culture flask was set to 0, 80 or 160 rpm during IPTG induction and the resultant expression levels of AspT were compared. No significant difference was observed between 0 rpm shaking and 80 rpm shaking (Fig 1B); however, when we compared the *P* values, we found that the expression level of AspT tended to be higher with 80 rpm shaking than with 0 rpm shaking (the 0 rpm/80 rpm *P*-value was 0.12 after a Bonferroni correction). In addition, the cell yield from this culture condition was higher than that from static (0 rpm) conditions (Fig. 1C). Therefore, we selected 80 rpm shaking for the subsequent assays. The high cell yield was observed with *E. coli* C43 (DE3) pLysS/pTrcAspD/T-His (data not shown), whereas with *E. coli* XL3/pTrcAspD/T-His cells lysed under conditions of 80 rpm of shaking no cell yield was obtained (Fig. 1C).

**Fig. 2:**
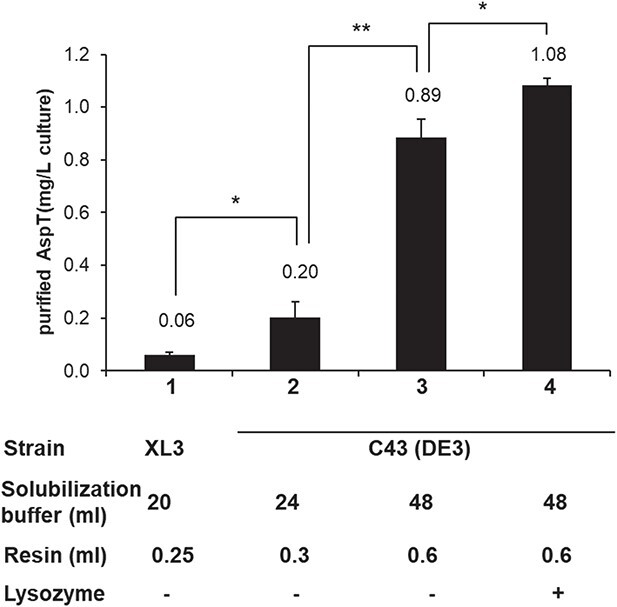
**Optimization of the AspT purification procedure.** Purification of AspT was carried out by the previously reported method of Sasahara *et al*. (5). The AspT yield from a 1-l culture of AspT-expressing XL3 cells under static conditions (control) is shown in column 1. In columns 2–4, AspT was purified from a 1-l culture of AspT-expressing C43(DE3) cells cultured under shaking (80 rpm) conditions. In column 3, the amount of solubilization buffer and TALON metal affinity resin used during the purification steps was increased. Lysozyme was added before cell disruption in column 4. Data are presented as the means of three independent experiments. Error bars indicate S.D. Statistical significance was analysed with Student’s paired *t*-test (^*^P < 0.05, ^**^P < 0.01 versus control group).

Next, to assess the production yields of the purified protein, purification yields of AspT from *E. coli* XL3/pTrcAspD/T-His cells and *E. coli* C43 (DE3)/pTrcAspD/T-His cells were compared. Protein yields from *E. coli* XL3/pTrcAspD/T-His cells or *E. coli* C43 (DE3)/pTrcAspD/T-His cells were 0.079 ± 0.013 μg/l culture or 0.277 ± 0.072 μg/l culture, respectively (Fig. 2, columns 1 and 2). The purified AspT from both strains showed almost identical levels of counter-flow activity in proteoliposomes (data not shown). To establish a method for efficient AspT purification from *E. coli* C43 (DE3)/pTrcAspD/T-His cells, the amount of DDM buffer needed during the solubilization step and the amount of TALON metal affinity resin needed in the purification procedure were assessed (Fig. 2, column 3). Increasing the ratio of solubilization buffer (from 24 to 48 ml/membrane in a 1-l culture) at the solubilization step and of TALON metal affinity resin (from 0.3 to 0.6 ml/solubilized protein in a 1-l culture) at the purification step led to higher protein yields (1.146 ± 0.049 mg). Moreover, addition of 1 mg/ml lysozyme to the cell suspension buffer before cell disruption enhanced cell lysis and increased the yield of purified AspT (Fig. 2, column 4).

**Fig. 3:**
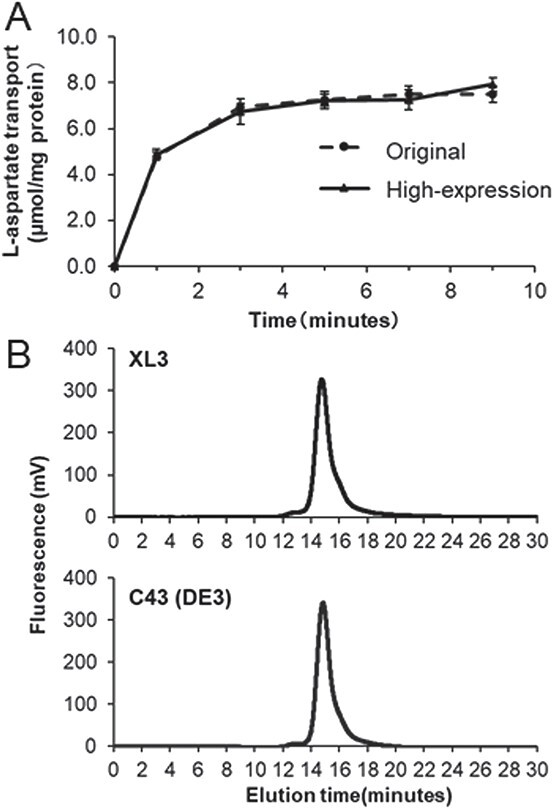
**Transport ability and molecular size of AspT purified by the optimized purification methods.** Purified AspT from AspT-expressing XL3 cells (broken line in (A) and upper panel in (B)) or AspT-expressing C43(DE3) cells (solid line in A and lower panel in B) was used for the L-Aspartate transport assay (A) and size-exclusion chromatography (B). (A) The transport assay was carried out using proteoliposomes as follows. Purified AspT was reconstituted into liposomes and radiolabelled L-Aspartate was added. To estimate L-Aspartate transport, aliquots were taken for filtration and washing at the times indicated. Radiolabelled L-Aspartate transport into proteoliposomes was measured by using a liquid scintillation counter. Data are presented as the means of three independent experiments. Error bars indicate S.D. (B) A total of 0.05 μg of purified AspT was ultracentrifuged and analysed by size-exclusion chromatography with a Superdex 200 Increase 10/300 GL column (Cytiva, Marlborough, MA, USA). The running buffer comprised 50 mM HEPES-HCl (pH 7) containing 0.01% DDM, 500 mM NaCl and 200 mM L-Aspartate (K-salt); the flow rate was 0.8 ml/min. Tryptophan fluorescence (Ex, 325 nm; Em, 280 nm) of the eluate was monitored using a fluorescence detector.

Next, we confirmed the substrate transport ability and molecular size of the purified AspT from *E. coli* C43 (DE3)/pTrcAspD/T-His cells by assessing counter flow with purified AspT reconstituted in proteoliposomes and size-exclusion chromatography, respectively. In the transport assays, radio-labelled L-aspartate was added to reaction buffer that contained purified AspT reconstituted in proteoliposomes. The amount of [^3^H]-L-Asp transported into the proteoliposomes was measured with a liquid scintillation counter. AspT purified from *E. coli* C43 (DE3)/pTrcAspD/T-His cells showed almost the same substrate transport ability as purified AspT from *E. coli* XL3/pTrcAspD/T-His cells (Fig. 3A). In the size-exclusion chromatography, the purified AspT was applied to a Superdex 200 Increase 10/300 GL column (Cytiva) and the tryptophan fluorescence was detected by using a fluorescence detector (L-7485). AspT purified from *E. coli* C43 (DE3)/pTrcAspD/T-His cells showed a single peak at 14.8 min with an almost symmetric shape (Fig. 3B, lower panel) that was consistent with the chromatographic findings with AspT purified from *E. coli* XL3/pTrcAspD/T-His cells. From these results, we conclude that the products of our optimized AspT overexpression and purification system maintained their transport ability and authentic molecular size.

**Fig. 4:**
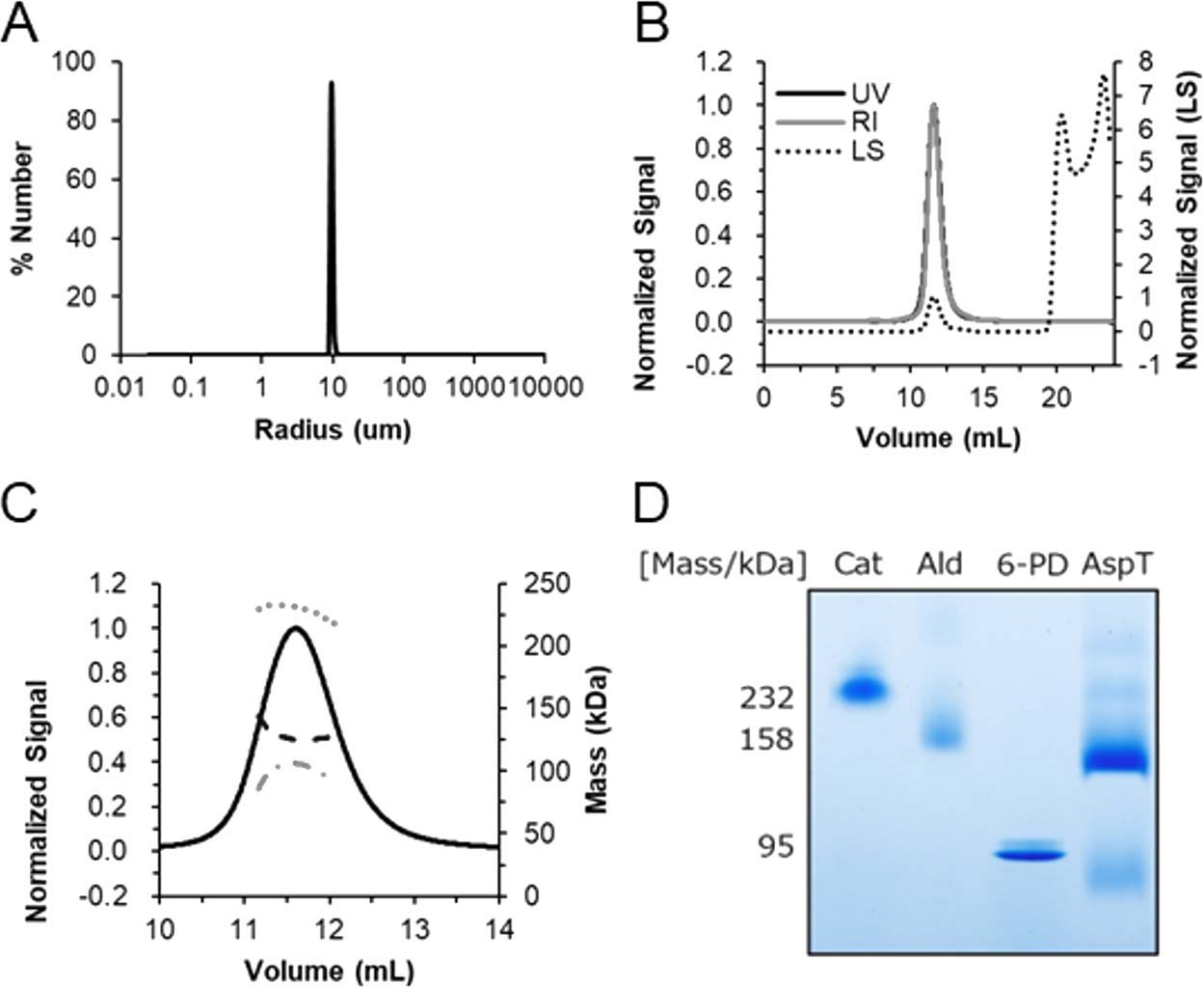
**Size distributions, SEC-MALS and BN-PinvitooAGE analysis of purified AspT.** (A) DLS of purified AspT in DDM. The experiment was conducted three times independently. A representative result is shown. (B) SEC-MALS of purified AspT in DDM. The chromatogram shows the normalized absorbance at UV 280 nm (UV) (black solid line) hidden by the normalized refractive index (RI) signal (grey solid line) and overlaid with the light scattering (LS) signal (black dotted line). (C) The absolute molecular mass of purified ApsT in DDM from SEC-MALS. The horizontal lines display the weight-averaged molar mass of the AspT-DDM complex (grey dotted line), AspT (black dashed line), DDM micelle (grey dash/dot line) and the UV (black solid line). The experiment was conducted three times independently. (D) BN-PAGE of purified AspT in DDM. AspT was solubilized in DDM and 2.8 μg was separated, using 2 μg catalase (232 kDa), 2 μg aldolase (158 kDa) and 3.4 μg 6-phosphogluconate dehydrogenase (95 kDa) as markers.

DLS analysis, which is used to investigate the monodispersity of purified proteins (17, 18), measures particle size by measuring the velocity of motion of nanoparticles in solution. We used DLS to determine the distribution of particle sizes of purified AspT in DDM, and a single size distribution at 9.9 ± 0.2 nm was revealed (Fig. 4A). The results showed that the purified AspT had a uniform molecular diameter.

SEC-MALS, which is used to measure the absolute molecular weight of proteins, combines gel filtration chromatography with MALS as a means of measuring the absolute molecular weight of particles exhibiting a single peak (18, 19). We used SEC-MALS to determine the absolute molecular mass of purified AspT in DDM at UV 280 nm absorbance and found a single peak (Fig. 4B) at a molecular mass of 229 kDa. We used the molar absorbance coefficient of AspT (calculated from its amino acid sequence as 0.456) to estimate the absolute molecular mass of AspT as 128 kDa (Fig. 4C). The theoretical mass of an AspT monomer, as calculated from its amino acid sequence, is 58 kDa; so from these SEC-MALS results, the purified AspT in DDM was predicted to be dimers, and the amount of DDM bound to the AspT dimer was predicted to be 110 kDa.

To further confirm the oligomeric status of AspT, purified AspT was subjected to BN-PAGE. In BN-PAGE, the undenatured protein is given a charge by binding to G-250 and electrophoresis can be performed while maintaining the complex (20, 21). Because the mobility of proteins on BN-PAGE depends mainly on their pI, marker proteins that have similar pI values to AspT (pI = 8.23) were selected as follows: catalase (232 kDa, pI = 8.39), aldolase (158 kDa, pI = 8.31) and 6-phosphogluconate dehydrogenase (95 kDa, pI = 8.39). The band of AspT was observed between 95 and 158 kDa (Fig. 4D). Since the theoretical mass of AspT is 58 kDa, the purified AspT is predicted to form dimers, and this prediction is in good agreement with the result of the SEC-MALS described above.

**Fig. 5:**
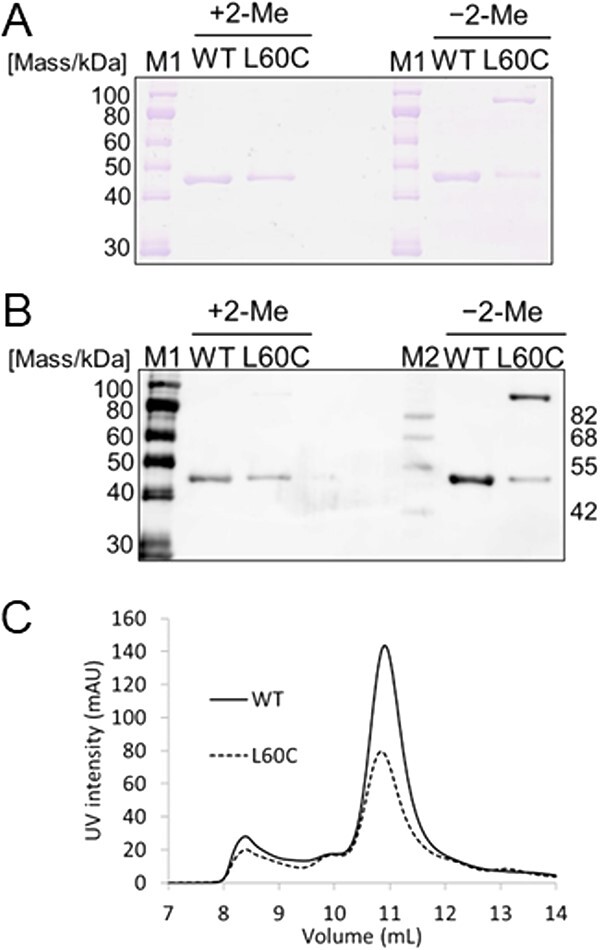
**SDS-PAGE, western blot and SEC analysis of WT and L60C mutant AspT.** (A) SDS-PAGE of AspT WT and L60C mutant. Purified proteins (0.5 μg of each) were electrophoresed under either reducing (+2-Me) or non-reducing (−2-Me) conditions. Numbered arrows give the mass of the standards in kDa. (B) western blot analysis of AspT WT and L60C mutant under either a reducing (+2-Me) or a non-reducing (−2-Me) condition. Purified proteins (0.5 μg of each) were subjected to western blotting with an anti-histidine-tag antibody. (C) Analytical SEC of AspT WT and L60C mutant. The purified AspT WT and L60C mutant were subjected to gel filtration chromatography. The chromatogram shows the intensity of 280 nm absorbance for AspT WT (black solid line) and L60C mutant (black dotted line). The experiment was conducted three times independently.

In a previous study, we suggested that AspT formed oligomers by crosslinking with glutaraldehyde and reported that when purified single-Cys mutants were subjected to SDS-PAGE under non-reducing conditions, a band was detected at the dimeric position (6). Since AspT has activity in the presence of the reducing agent DTT (3) and replacing three endogenous Cys residues of AspT with Ala does not affect its transport activity (6), we presumed that the endogenous Cys does not form disulphide bonds and is not involved in the function. In this study, we focused on one of these mutants, the AspT L60C mutant. This mutant replaced three AspT endogenous Cys with alanine, and then replaced the leucine 60 with a Cys. First, the L60C mutant was purified and subjected to SDS-PAGE and western blot. The WT showed a single band at ~45 kDa (the monomeric position), regardless of non-reducing or reducing conditions. However, the L60C mutant under the non-reducing condition showed a band at the dimeric position of about 100 kDa on gels, indicating an inter-molecular disulphide bond between the introduced Cys residues (Fig. 5A and B). The results were consistent with our previous report (6).

Based on the above results, we estimated that a peak at the dimeric position would be observed when purified L60C was subjected to gel filtration. Purified WT and purified L60C were subjected to gel filtration chromatography, and the positions of the peaks of both purified proteins were compared. In Fig. 5C, both proteins showed similar peaks around the elution volume of 11 ml. Taken together, these data are strong evidence that AspT forms a dimer.

## Discussion

Analysis of protein oligomeric structures requires large amounts of purified proteins, but membrane proteins are generally highly toxic and difficult to express in high yields; for example, the yield of AspT by previous methods was 0.06 mg/l culture (Fig. 2, column 1). Because the lack of highly purified AspT has prevented further studies on the detailed oligomeric structure of AspT, we first optimized the expression and purification conditions of AspT. Optimization of the *E. coli* strains, the culture conditions and the purification conditions yielded sufficient protein (1.08 mg of purified protein from 1 l of culture) (Figs 1 and 2).

We then confirmed that the protein purified by our modified protocols had the same molecular weight, purity and functions as the protein purified by the original protocol (Fig. 3). *Escherichia coli* C43 (DE3) is highly resistant to toxins, which may have increased the stability of expression of even highly toxic membrane proteins (22). As for the shaking speed, increasing the shaking speed is generally expected to increase the yield due to better aeration, but since AspT is highly toxic, the high expression of AspT under 160 rpm shaking caused the bacteria to lyse, which decreased the yield. At 80 rpm, the expression was suppressed enough for the bacteria to be spared from lysis, and thus production was increased. As for the purification method, the addition of lysozyme enabled efficient disruption of the bacteria with progressive lysis of the cell wall, which may have increased the recovery rate of purified AspT.

SEC-MALS and BN-PAGE analyses suggest that AspT solubilized in DDM formed a dimer (Fig. 4B–D), and the purified AspT mutant L60C was found to have a band at the dimeric position under non-reducing conditions (6) (Figs 5A and B). These results suggest that the L60C residue was located in a region where the dimers are close to each other and can be purified while maintaining SS binding. Therefore, the elution position in SEC of the purified L60C mutant was expected to be the dimeric position. The results of this experiment show that the elution positions of WT and L60C were similar (Fig. 5C), which strongly suggests that the purified AspT WT in the solubilized state is also a dimer.

The present study, which analysed the oligomeric structure of AspT by means of SEC-MALS and BN-PAGE and then compared the elution time in gel filtration chromatography of AspT WT and the L60C mutant, suggests that AspT forms dimers. Transporters are known to form a variety of oligomeric structures and to employ several different mechanisms in the transport processes of substrates (23). For instance, the major facilitator super-family transporters LacY, MelB and OxlT are functional as monomers (24–26), whereas the Na^+^/H^+^ antiporter NhaA forms dimers, and the functional units of the glutamate transporter Gltph are trimers (27, 28). Structural and biochemical studies are necessary to clarify the transport mechanism of AspT.

## Author contributions

A.M., K.N. and K.A. designed the study. A.M., T.Y. and K.K. performed the experiments and analysed the results. A.M., T.Y., K.N. and K.A. prepared the manuscript. All authors discussed the results.
